# Supplementary material for: Association between dietary intake of flavonoid and chronic kidney disease in US adults: Evidence from NHANES 2007-2008, 2009-2010, and 2017-2018
Source: PLoS One. 2024 Aug 27;19(8):e0309026. doi: 10.1371/journal.pone.0309026 (PMC11349111; doi:10.1371/journal.pone.0309026)
Supplement: Attachment — Submitted filename: Response to Reviewers.docx [file pone.0309026.s001.docx]

**Dear editors and reviewers:**

Thank you for your letter and for reviewers' constructive comments on my manuscript entitled “Association between dietary intake of flavonoid and chronic kidney disease in US adults: Evidence from NHANES 2007-2008, 2009-2010, and 2017-2018” (Manuscript ID: PONE-D-24-13778). Those comments are all valuable and helpful for revising and improving our paper. We have carefully considered the suggestion of Reviewers and have made some corrections. Revised portion are marked in red in the paper. The main corrections in the paper and the responds to the reviewers' comments are as flowing:

**Reviewer #1:**

It is a very interesting article. It is well-designed although cross-sectional. However, I think a professional should review the statistical analyses. The figures are vague. It would be nice to analyze the effects according to different stages of CKD, both GFR stages and albuminuria stages.

**Response to reviewers:**

Thank you for your positive feedback and valuable suggestions. We appreciate your insights and have made the following revisions and additions to address your concerns.

1.**Statistical Analyses:** we have conducted a thorough review of the statistical analyses with the assistance of a professional statistician to ensure accuracy and robustness.

2.**Clarity of Figures:** we acknowledge that the figures could be clearer. To address this, we have revised the figures to improve their clarity and readability. The updated figures are included in the revised manuscript.

3. **Analysis by CKD subgroups**: after adjusting for variables including age, sex, body mass index, ethnicity, smoke status, energy intake, physical activity status, hyperuricemia, hyperlipidemia, hypertension, and diabetes mellitus (Table 3), the odds ratios (ORs) for decreased eGFR risk in the Q2, Q3, and Q4 groups of total flavonoid intake, compared with that for decreased eGFR incidence in the Q1 group, were 0.529 (95% CI: 0.341, 0.821), 0.354 (95% CI: 0.237, 0.529), and 0.533 (95% CI: 0.329, 0.863), respectively. The P value for the trend test was 0.046. The results suggested that higher intake of dietary flavonoid is associated with a significantly lower risk of decreased eGFR, with the most substantial reduction observed in Quartile 3. In contrast, there is no significant association between flavonoid intake and the risk of elevated uACR. The trend for decreased eGFR indicates that as flavonoid intake increases, the risk decreases. However, the risk of elevated uACR does not show a significant trend with increasing flavonoid intake across the quartiles.

Table 3 Association between dietary flavonoid and decreased eGFR/increased uACR in the US population

|  |  | **OR (95%CI)** | | | | **P value for trend** |
| --- | --- | --- | --- | --- | --- | --- |
| Outcomes | Total flavonoid  (mg per day) | Q1  [0, 20.65] | Q2  (20.65, 49.74 | Q3  (49.74, 140.15] | Q4  (140.15, 9969.83] |  |
| Decreased eGFR | | ref | **0.529 (0.341,0.821)** | **0.354 (****0.237,0.529)** | **0.533 (0.329,****0.863)** | **0.046** |
| Elevating uACR | | ref | 0.980 (0.686,1.399) | 1.072 (0.791,1.453) | 0.891 (0.676,1.173) | 0.410 |

Abbreviations: OR, odds ratio; CI, confidence interval; Q1, quartile 1; Q2, quartile 2; Q3, quartile 3; Q4, quartile 4.

Model 1 did not include any covariate. Model 2 was adjusted for age and sex. Model 3 was adjusted for age, sex, body mass index, ethnicity, smoke status, energy intake, physical activity status, hyperuricemia, hyperlipidemia, hypertension, and diabetes mellitus.

Changes in lines: 93-100, 166-171.

**Reviewer #2:**

1.The article aims to find an association between dietary intake of flavonoid and CKD in US adults using the NHANES data between 2007-2008. 2009-2010 and 2017-2018. The methodology is complex and requires more explanation. For instance: in lines 53-55 the authors write "This study employed a cross-sectional design and used data from three cycles of the continuous NHANES: 2007–2008, 2009–2010, and 2017–2018. After weighting, the findings of this study are representative of 306,640,845 US residents". "Weighting'' needs to be explained clearly so the reader is left with a clear understanding of the methodology. This also applies to line 135 of the manuscript "Post-weighting, the data in this study represented 213,259,068 US adults aged 20 years..." . ''Post-weighting'' should also be clearly explained.

**Response to reviewers:**

Thank you for your valuable feedback and for pointing out the need for further explanation regarding the methodology, especially the concepts of "weighting" and "post-weighting." We have revised the manuscript to provide a clearer understanding of these terms and their application in our study. Below is our detailed response and the changes made to the manuscript.

Weighting is a crucial step in the analysis of NHANES data. It involves adjusting the data to account for the survey's complex sampling design, including oversampling of certain populations, survey non-responses, and post-stratification to match the US population demographics. This process ensures that the results can be generalized to the entire US population. The weighting factor provided by NHANES is applied to each participant to make the sample representative of the broader population. We have incorporated these clarifications into the manuscript to enhance the understanding of the weighting process and its importance in making our findings representative of the US population. Additionally, we have created a flowchart of the population selection, as shown in Figure 1. We believe these changes address your concerns and improve the overall clarity of the methodology section.

Changes in Lines: 53-58, 146-150.

2. The text in lines 163-165: "Among these subgroups, all trend tests yielded P < 0.05, except for participants older than 65 years and those with hyperlipidemia. Interaction tests within these subgroups also exhibited P > 0.05". Table 3 does not align with the text here, adults >65 years had a p-value for trend of 0.059 and a p-value for interaction of 0.856. Those with hyperlipidemia also had a p-value >0.05. Please clarify these seeming discrepancies. Also review lines 167-168 and Table 4 to ensure the text aligns with the table.

**Response to reviewers:**

Thank you again for your thorough review and valuable comments.

We have revised the manuscript to accurately reflect the statistical results, ensuring consistency between the text and Tables 4 and 5. These changes address the discrepancies noted and improve the clarity and accuracy of our findings.

Changes in Lines:182-192.

**Reviewer #3:**

1.The authors do not clearly state the primary outcome in the Methods section. The reader cannot understand what the authors are trying to clarify.

**Response to reviewers:**

Thank you for your feedback regarding the clarity of the primary outcome in the Methods section. We appreciate your attention to detail.

We apologize for the oversight and have revised the Methods section to clearly state the primary outcome of our study. The primary outcome we aimed to investigate is the association between dietary flavonoid intake and CKD risk in US adults.

Changes in Lines: 93-100.

2. The authors' data collection method is very vague, and it is difficult to determine whether a quantitative evaluation can be made with a questionnaire. The authors' method of converting disparate data into continuous variables and then calculating averages from them is not appropriate.

**Response to reviewers:**

Thank you for your feedback. We understand the concerns raised regarding the data collection method and the subsequent data analysis.

**Clarification of Data Collection Method:** The National Health and Nutrition Examination Survey (NHANES) is a program designed to assess the health and nutritional status of adults and children in the United States. The survey is unique because it combines interviews with physical examinations. NHANES is a major program of the National Center for Health Statistics (NCHS), which is part of the Centers for Disease Control and Prevention (CDC). NCHS is responsible for producing vital and health statistics for the nation. This study involved a secondary analysis of NHANES data. We did not participate in the data collection or cleaning processes. Importantly, NHANES data underwent stringent quality control measures before being released.

**Appropriateness of Converting Disparate Data into Continuous Variables:** We appreciate your concern regarding our data analysis methods. Upon review, we realize there may have been some confusion. Specifically, we did not convert categorical variables into continuous variables and then calculate their means. Instead, we treated the data according to its original format and used appropriate statistical methods for analysis. For categorical data, we employed frequency and proportion calculations, and for continuous data, we used measures as means and with adjusted 95% confidence interval.

3. Except when consuming nutrient extracts such as supplements, nutrients obtained from food are complex and interact, making it difficult to consider some nutrients obtained from food in isolation. It is difficult to rule out various confounding factors, such as alcohol in wine or caffeine in tea.

**Response to reviewers:**

Nutrients obtained from food are complex and interact with one another, which poses challenges when considering individual nutrients in isolation, except when consuming nutrient extracts such as supplements. It is difficult to rule out various confounding factors, such as the presence of alcohol in wine or caffeine in tea. These confounders might influence outcomes and must be carefully considered and controlled for in our analysis. However, we acknowledge that it is not possible to account for all potential confounding factors. Therefore, we selected some classic confounding factors, such as sex, age, smoking status, diabetes, and hypertension. The relationships between caffeine, alcohol, and CKD risk are still a subject of debate, so we did not include these variables as adjustments in our analysis.

4. It is difficult to dispel the bias that people who consume more flavonoids are more health conscious. Therefore, it is difficult to prove causality outside of intervention studies.

**Response to reviewers:**

Thank you for your feedback. We acknowledge the concern that individuals who consume more flavonoids may generally be more health-conscious, which can introduce bias. This makes it challenging to prove causality outside of intervention studies.

To establish a more robust causal relationship between flavonoid intake and CKD risk, further foundational research and cohort studies are necessary. These studies would help to validate and clarify the potential causal links between flavonoid intake and CKD.

We have taken this into consideration in our analysis and have discussed this limitation in the Discussion section of our study. While we have controlled for various confounding factors, such as sex, age, smoking status, diabetes, and hypertension, we recognize that residual confounding may still exist.

Changes in Lines: 273-275.

5.The resolution of Fig. 2 is poor.

**Response to reviewers:**

Thank you for your feedback regarding Figure 3. We apologize for the poor resolution of the figure.

We will provide an updated version of Figure 3 with improved resolution to ensure clarity and better visualization of the data. This updated figure will be included in the revised manuscript.

**Reviewer #4**:

1. The study population: In Methods, lines 55 and 57, the study is said to represent 306,640,845 US residents after weighting, and after exclusion of individuals under 20 years and those with missing data, the total is 13,259,068. On the other hand, in Results, line 135 the number is 213,259,068 US adults >20 years of age. Please comment on this discrepancy, and also provide the actual number of participants, ie before the weighting.

**Response to reviewers:**

Thank you for pointing out the discrepancy in the reported study population. We apologize for any confusion this may have caused.

In the Methods section (lines 55 and 57), the study initially represented 306,640,845 US residents after weighting. However, after excluding individuals under 20 years of age and those with missing data, the weighted total was 213,259,068 US adults. The discrepancy in the Results section (line 135) where the number is reported as 13,259,068 is an error.

To clarify, the actual number of participants before weighting was 29,940. After applying the weights to account for the complex survey design and ensure national representativeness, the estimated population was 213,259,068 US adults excluding those less than 20 years old and having missing values.

We will correct this discrepancy in the revised manuscript to ensure consistency and accuracy. Additionally, we will include a flowchart (Figure 1) to illustrate the population selection process for better understanding.

Changes in line:146-150.

2. The outcome variable is CKD, based on an e-GFR of <60 ml/min/1.73m2 or as UAC of >30 mg/g. According to these criteria, 14.5% of the study population has CKD. In recent years, studies have shown that this may overestimate the prevalence of CKD, and that age-adapted e-GFR may be more appropriate and would consider the physiologic age-related decline in GFR. Overestimation of eGFR in the elderly could bias the outcome of the study. Please comment on this.

**Response to reviewers:**

I agree with your perspective that using an e-GFR of <60 ml/min/1.73m² or a UAC of >30 mg/g to define CKD can easily lead to an overestimation of CKD prevalence, especially in cross-sectional studies. This discrepancy arises from various factors, including the reliance on single measurements of kidney function, which can be influenced by temporary conditions like dehydration or acute illness. Additionally, certain population-based screening methods may not account for age-related declines in kidney function that do not necessarily indicate disease. As a result, there is a growing consensus that more refined and repeated measures are necessary to accurately assess CKD prevalence, ensuring that public health interventions are appropriately targeted.

Compared to estimating GFR, measuring GFR is more accurate but also more expensive and complex, making it impractical for routine clinical use. GFR estimation equations, on the other hand, offer the advantages of accuracy, convenience, and speed. There are currently over a hundred GFR estimation equations, with the most recognized being the MDRD (Modification of Diet in Renal Disease) equation, the CKD-EPI (Chronic Kidney Disease Epidemiology Collaboration) equation, and the Cockcroft-Gault equation. This study used the 2012 CKD-EPI equation, which was widely accepted and recommended by the KIDGO guidelines. However, the 2012 CKD-EPI equation has limitations in various medical scenarios, such as in patients with diabetes, liver cirrhosis, and the elderly.

Overall, the 2012 CKD-EPI equation based on serum creatinine is suitable for most clinical situations, with GFR measurement considered in special cases.

3. I propose that the authors describe the types of food and beverages that belong to the various subclass of flavonoids in a table. This will give the reader a better picture of the results.

**Response to reviewer:**

Thank you for your valuable suggestion. We agree that providing information about the types of food and beverages belonging to various subclasses of flavonoids would enhance the clarity of our results. According to data released by the United States Department of Agriculture, a variety of foods have been identified as rich sources of flavonoids, including beans, bee pollen, broad beans, coriander, cress, dill weed, fennel, hartwort, and onions, among others [1]. However, due to the extensive number of food and beverage items containing flavonoids, it is challenging to list them all comprehensively.

Instead of including a detailed table, we have provided a link where readers can find a comprehensive list of flavonoid-rich foods and beverages. This approach ensures that readers have access to detailed information without overwhelming the manuscript. Specific flavonoid-rich foods can be found in the references provided.

1.	David B. Haytowitz, X.W., and Seema Bhagwat. *2018. USDA Database for the Flavonoid Content of Selected Foods, Release 3.3.*; Available from: <http://www.ars.usda.gov/nutrientdata/flav>.

4. There is a great variation in the intake of various subtypes of flavonoids (table 4). Flavonols are abundant and the only subtype that has a significantly negative association with CKD. Flavan-3-ols are also abundant in the food but do not confer renal protection. Comment on that please. In Discussion, lines 253-254, the authors say: “the excessive daily intake of flavan-3-ols may potentially affect the biological effects of other flavonoid subgroups”. How? Has this to do with the J shaped curve?

**Response to reviewers:**

**Part 1**: Thank you for your insightful comment. We appreciate the opportunity to elaborate on the observed variations in the intake of different flavonoid subtypes and their associations with CKD, as presented in Table 4.

Indeed, flavonols are abundant in the diet and are the only subtype that has shown a significantly negative association with CKD. This finding suggests that flavonols may possess unique properties that confer renal protection. Several studies have indicated that flavonols, such as quercetin and kaempferol, have anti-inflammatory and antioxidant effects, which may play a role in mitigating the progression of CKD.

On the other hand, flavan-3-ols, despite being abundant in food sources such as tea, apples, and cocoa, do not demonstrate the same protective effect against CKD. This lack of association could be attributed to differences in the bioavailability, metabolism, and specific biological activities of flavan-3-ols compared to flavonols. While flavan-3-ols have been shown to have cardiovascular benefits, their impact on renal function may be less pronounced or mediated through different mechanisms that do not directly influence CKD progression.

On the other hand, flavan-3-ols, despite being abundant in food sources such as tea, apples, and cocoa, do not demonstrate the same protective effect against CKD. This lack of association could be attributed to differences in the bioavailability, metabolism, and specific biological activities of flavan-3-ols compared to flavonols. While flavan-3-ols have been shown to have cardiovascular benefits, their impact on renal function may be less pronounced or mediated through different mechanisms that do not directly influence CKD progression.

This discrepancy underscores the importance of understanding the distinct biological effects of various flavonoid subtypes. Further research is needed to elucidate the mechanisms underlying these differences and to explore the potential therapeutic benefits of specific flavonoid subtypes in CKD prevention and management.

**Part 2**: In the Discussion, lines 253-254, we mention that "the excessive daily intake of flavan-3-ols may potentially affect the biological effects of other flavonoid subgroups." This statement addresses the complex interactions that can occur between different types of flavonoids when consumed in large quantities.

Excessive intake of flavan-3-ols could potentially influence the absorption, metabolism, and overall bioavailability of other flavonoid subgroups. This interaction might occur due to competition for metabolic enzymes and transporters, which could alter the efficacy and biological activity of these compounds. Additionally, high concentrations of flavan-3-ols might exert an antioxidant load that could disrupt the redox balance, potentially diminishing the beneficial effects of other flavonoids.

Regarding the J-shaped curve, this concept suggests that while moderate intake of certain compounds may provide health benefits, excessive intake can lead to adverse effects. In the context of flavonoids, moderate consumption of flavan-3-ols might contribute to health benefits, but excessive consumption could lead to negative interactions with other flavonoid subgroups, reducing their efficacy or potentially leading to unintended adverse effects. This could partially explain why flavan-3-ols, despite their abundance, do not show a protective effect against CKD in our study, while flavonols do.

We hope this clarifies the potential mechanisms behind the interactions of flavonoid subgroups and the relevance of the J-shaped curve in this context. Thank you again for your insightful question.

5. In Discussion, line 218 and more, Quercetin is discussed and said to be flavanol, but should be flavonol.

**Response to reviewers:**

Thank you for pointing out this error. You are correct that quercetin is a flavonol, not a flavanol. We apologize for this oversight. We will correct this in the revised manuscript to ensure accuracy.

Changes in Lines: 239

Of note, the images we created were made according to PLOS ONE's requirements. However, they appear distorted after being uploaded to the system. I am not sure why this happens, but the reviewers will be able to see clear images after downloading them.

We appreciate the opportunity to improve our manuscript and are open to any further suggestions you may have.

Sincerely,

Peijia Liu and Guixia Li
